# Mumps virus Enders strain is sensitive to interferon (IFN) despite encoding a functional IFN antagonist

**DOI:** 10.1099/vir.0.013722-0

**Published:** 2009-11

**Authors:** D. F. Young, M. C. Galiano, K. Lemon, Y.-H. Chen, J. Andrejeva, W. P. Duprex, B. K. Rima, R. E. Randall

**Affiliations:** 1 Centre for Biomolecular Sciences, University of St Andrews, St Andrews, Fife KY16 9ST, UK; 2 Centre for Infection and Immunity, School of Medicine, Dentistry and Biomedical Sciences, The Queen's University of Belfast, 97 Lisburn Road, Belfast BT9 7BL, UK

## Abstract

Although the Enders strain of mumps virus (MuV) encodes a functional V protein that acts as an interferon (IFN) antagonist, in multi-cycle growth assays MuV Enders grew poorly in naïve (‘IFN-competent’ Hep2) cells but grew to high titres in ‘IFN-compromised’ Hep2 cells. Even so, the growth rate of MuV Enders was significantly slower in ‘IFN-compromised’ Hep2 cells when compared with its replication rate in Vero cells and with the replication rate of parainfluenza virus type 5 (a closely related paramyxovirus) in both naïve and ‘IFN-compromised’ Hep2 cells. This suggests that a consequence of slower growth is that the IFN system of naïve Hep2 cells can respond quickly enough to control the growth of MuV Enders. This is supported by the finding that rapidly growing variants of MuV Enders that were selected on ‘IFN-compromised’ Hep2 cells (i.e. in the absence of any selection pressure exerted by the IFN response) also grew to high titres on naïve Hep2 cells. Sequencing of the complete genome of one of these variants identified a single point mutation that resulted in a substitution of a conserved asparagine by histidine at position 498 of the haemagglutinin–neuraminidase protein, although this mutation was not present in all rapidly growing variants. These results support the concept that there is a race between the ability of a cell to detect and respond to virus infection and the ability of a virus to block the IFN response. Importantly, this emphasizes that factors other than viral IFN antagonists influence the sensitivity of viruses to IFN.

## INTRODUCTION

Cells respond to virus infection by secreting alpha and beta interferons (IFN-*α*/*β*), which act in both autocrine and paracrine fashions to upregulate the expression of hundreds of cellular genes, the products of many having antiviral functions. Cells detect the presence of viruses by having receptors that recognize specific pathogen-associated molecular patterns (PAMPs). PAMPs are often viral nucleic acids, produced during the virus replication cycle (reviewed by Randall & Goodbourn, 2008; Takeuchi & Akira, 2009). RIG-I and mda-5 are two intracellular detectors of viral PAMPs which recognize RNA structures not normally present in cells, such as double-stranded RNA (dsRNA) and/or 5′-triphosphorylated, uncapped single-stranded RNA (ssRNA) (Andrejeva *et al.*, 2004; Yoneyama *et al.*, 2004; Hornung *et al.*, 2006; Pichlmair *et al.*, 2006; Kato *et al.*, 2008). When activated by their appropriate ligands, RIG-I and mda-5 initiate a signalling cascade that ultimately leads to the synthesis of IFN-*α*/*β*. Secreted IFN-*α*/*β* binds to the IFN-*α*/*β* receptor and activates JAK1 and Tyk2, two kinases associated with the cytoplasmic domain of the receptor. These in turn phosphorylate the latent cytoplasmic transcription factors STAT1 and STAT2, which then form stable heterodimers and associate with IRF-9 to create the ISGF3 transcription factor complex that activates IFN-*α*/*β* responsive genes (reviewed by Platanias, 2005; Randall & Goodbourn, 2008).

Many of the genes upregulated by IFN encode proteins that have either direct or indirect antiviral activities that can limit virus replication. Although their antiviral activities are often restricted to specific viruses or virus families, there are examples of IFN-inducible antiviral proteins that act at most stages of the growth cycle of viruses, from entry and uncoating (e.g. Trim5*α*), to viral replication and transcription (e.g. Mx), viral protein synthesis (e.g. PKR and oligo A synthetase) and virus egress (e.g. viperin and tetherin) (reviewed by Randall & Goodbourn, 2008). However, viruses can still replicate and cause disease *in vivo* because they encode products, usually proteins, which interfere with some aspect of the IFN response. Nevertheless, viruses are usually incapable of completely circumventing the IFN response, which remains critical in restricting virus replication during the initial stages of virus infection prior to development of adaptive immune responses. Over the last few years, a great deal has been learnt about the mechanisms of action of many viral IFN antagonists (reviewed by Haller *et al.*, 2006; Randall & Goodbourn, 2008). Loss of function of viral IFN antagonists sensitizes viruses to the IFN response, such that the viruses cannot replicate in IFN-competent cells and are non-pathogenic in IFN-competent animals. However, such viruses usually replicate well in IFN-compromised cells and remain pathogenic in IFN-compromised animals. Although the critical importance of viral IFN antagonists in facilitating virus replication in IFN-competent cells is without dispute, it is not clear whether other virus–host interactions can influence the sensitivity of viruses to the IFN response.

*Mumps virus* (MuV) is a rubulavirus within the family *Paramyxoviridae*, a group of negative-sense ssRNA viruses (reviewed by Lamb & Parks, 2006). Like other rubulaviruses, MuV encodes an IFN antagonist termed the V protein. As with its close relative parainfluenza virus type 5 (PIV5; formerly known as SV5; Chatziandreou *et al.*, 2004), the V protein of MuV targets STAT1 for proteasome-mediated degradation, thereby blocking IFN signalling (Didcock *et al.*, 1999; Kubota *et al.*, 2001; Nishio *et al.*, 2002; Yokosawa *et al.*, 2002). MuV V protein also targets STAT3 for degradation, but it is unclear what biological role this plays during infection (Ulane *et al.*, 2003). This V function is separate from its role in STAT1 degradation (Puri *et al.*, 2009). In addition, the V proteins of MuV, PIV5 and most other paramyxoviruses can help limit IFN production by binding and inhibiting mda-5 (Andrejeva *et al.*, 2004; Childs *et al.*, 2007). Mumps is a serious human illness whose symptoms often include meningitis. Consequently, vaccination against MuV is routine (Plotkin, 2004). The Enders strain of MuV was isolated in 1945 (Enders *et al.*, 1946) and has subsequently been widely used for laboratory studies on MuV. In a neonatal hamster model (used to compare the pathogenicity of different MuV isolates), MuV Enders is highly attenuated in comparison with wild-type viruses (McCarthy *et al.*, 1980). Here, we report that the Enders strain of MuV is sensitive to the human IFN system even though it encodes a functional IFN antagonist.

## METHODS

### Cells, viruses and plasmids.

Vero, Hep2, 293 and A549 cells (and derivatives) were grown as monolayers in 25 cm^2^ or 75 cm^2^ tissue culture flasks, in Dulbecco's modified Eagle's medium supplemented with 10 % (v/v) fetal calf serum at 37 °C. PIV5 (strain W3) (Choppin, 1964) and MuV Enders (Enders *et al.*, 1946) virus stocks were grown and titrated (in duplicate) under appropriate conditions in Vero cells. The construction and properties of Hep2/BVDV-Npro and Hep2/PIV5-V cells have been previously described (Hilton *et al.*, 2006; Carlos *et al.*, 2007). The generation of Hep2/MuV-V cells was as previously described for Hep2/BVDV-Npro cells. Basically, the V gene was PCR amplified from MuV Enders-infected cells, cloned into a modified self-inactivating, bicistronic, lentiviral expression vector derived from pHR-SIN-CSGW (Demaison *et al.*, 2002), and used to generate recombinant lentivirus particles that were used to select for cells that express V5-tagged MuV-V as previously described (Hilton *et al.*, 2006).

### Selection of MuV Enders on naïve Hep2 and Hep2/BVDV-Npro cells.

Naïve Hep2 cells were infected at an m.o.i. of 1 p.f.u. per cell and after inoculation, the infected monolayer was trypsinized and cells were seeded into fresh flasks with a 10-fold excess of uninfected naïve Hep2 cells. After stationary incubation overnight at 37 °C, confluent monolayers were incubated on a rocker for a further 1–2 days. Cells were trypsinized and reseeded with a further 10-fold (for the first three passages) or 100-fold (for a further two passages) excess of uninfected naïve Hep2 cells and incubated as above. Four days after the fifth passage, the medium was harvested, titrated and used to inoculate Vero monolayers in 96-well plates at an m.o.i. of 0.1 p.f.u. per well. At 7–8 days after infection, medium was harvested from 30 wells that showed a cytopathic effect, and these were termed MuV Enders clone 3 subclones 1–30.

A selection of MuV Enders was made in naïve and BVDV-Npro-expressing Hep2 cells which followed basically the same regime as above (using MuV Enders clone 3 to inoculate, but diluting infected : uninfected cells 1 : 100 at every passage) for four passages. Two days after the fourth passage, medium was harvested and the amount of infectious virus was titrated by plaque assay.

### Immunoprecipitation, immunoblotting and immunofluorescence.

The procedures for immunoblotting, immunofluorescence and immunoprecipitation have been described previously (Randall & Dinwoodie, 1986; Carlos *et al.*, 2005). Antibodies used in these procedures included monoclonal antibodies (mAbs) to the V5-tag (Hanke *et al.*, 1992; Serotec, MCA1360), and polyclonal antibodies to STAT1 (Santa Cruz, sc-417), STAT3 (Abcam, ab2984) and *β*-actin (Sigma, A5441).

### Sequencing.

Naïve Hep2 cells were infected at an m.o.i. of 0.1 p.f.u. per cell. Two days post-infection, total RNA was prepared using TRIzol reagent (Invitrogen, 15596). RT-PCR was performed using superscript III one-step RT-PCR kit (Invitrogen, 12574) and gene-specific primers spanning the complete MuV genome (primer sequences are available on request). RT-PCR products were purified using GenElute PCR clean-up kit (Sigma, NA1020) and sequencing reactions were performed using Big Dye Terminator v3.1 cycle sequencing kit (Applied Biosystems, 4336917).

## RESULTS

### The Enders strain of MuV does not grow efficiently in IFN-competent Hep2 cells but grows to high titres in IFN-compromised Hep2 cells

**Fig. 1. f1:**
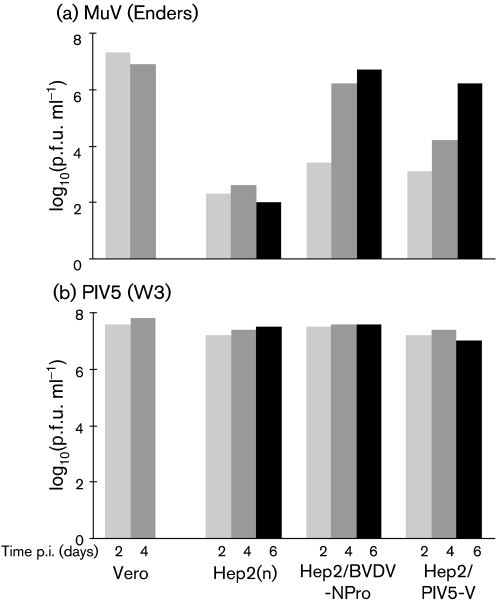
Multiple-step growth analysis of MuV Enders (a) and PIV5 (b). Vero cells, naïve Hep2 cells (Hep2n) and Hep2 cells that constitutively express either PIV5-V protein (which are unable to respond to IFN) or BVDV-Npro (which cannot produce IFN) were infected at an m.o.i. of 0.01 p.f.u. per cell, and the amount of infectious virus in the culture medium was titrated at 2, 4 and 6 days p.i. Note: similar experiments were carried out on multiple occasions and the results shown are representative of a typical experiment.

Whilst comparing the replication of a number of viruses in cells that can or cannot produce and respond to IFN, we noted that, unlike the closely related virus PIV5, the Enders strain of MuV did not replicate well in naïve Hep2 cells that can produce and respond to IFN (Fig. 1). However, MuV Enders grew to high titres in Hep2 cells engineered to be either unresponsive to IFN (Hep2/PIV5-V cells; Young *et al.*, 2003) or unproductive for IFN (Hep2/BVDV-Npro; Hilton *et al.*, 2006; Carlos *et al.*, 2007), illustrating that MuV is sensitive to the IFN response of Hep2 cells (Fig. 1). However, even in these ‘IFN-compromised’ Hep2 cells, MuV Enders had delayed growth kinetics since following a low m.o.i., the virus reached titres of 10^6^–10^7^ p.f.u. ml^−1^ at 4–6 days post-infection (p.i.), whereas in Vero cells [monkey cells which do not produce IFN due to spontaneous gene deletions (Desmyter *et al.*, 1968; Mosca & Pitha, 1986)], it had reached titres of 10^7^ p.f.u. ml^−1^ by 2 days p.i. Also in contrast with MuV Enders, PIV5 reached titres of 10^7^–10^8^ p.f.u. ml^−1^ in both ‘IFN-competent’ and ‘IFN-compromised’ Hep2 cells by 2 days p.i. (Fig. 1).

### MuV Enders V protein targets STAT1 and STAT3 for degradation and interacts with mda-5

**Fig. 2. f2:**
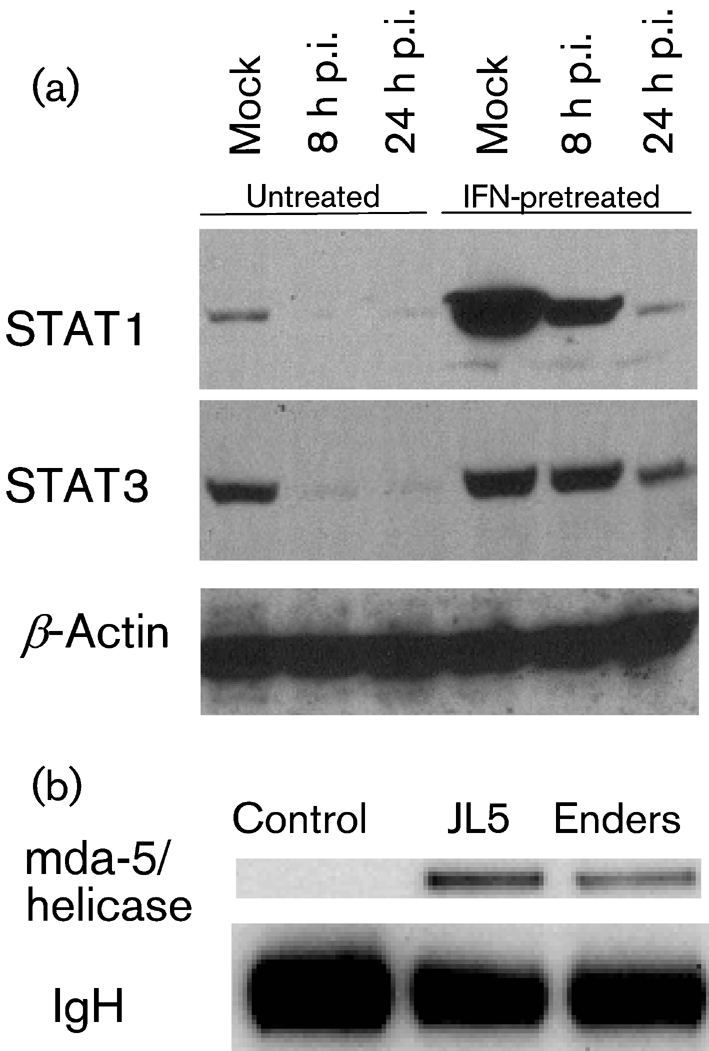
MuV Enders V protein targets STAT1 and STAT3 for degradation and interacts with mda-5. (a) Naïve Hep2 cells were infected with MuV Enders for 8 h, or mock infected, prior to addition of IFN-*α* [Roferon A (Roche) 1000 IU ml^−1^] to the culture medium. At 8 and 24 h p.i., cells were harvested and the presence of STAT1 and STAT3 was detected by immunoblot analysis. *β*-Actin acted as a loading control. (b) 293 cells were transfected with a control plasmid (empty vector) or plasmids expressing V5-tagged V proteins of MuV Enders or JL5, which has previously been shown to bind mda-5 (Andrejeva *et al.*, 2004; Childs *et al.*, 2007), together with a plasmid that expresses the FLAG-tagged helicase domain of mda-5. At 48 h post-transfection, the V proteins were immunoprecipitated and the presence of the mda-5 helicase domain was detected by immunoblot analysis. Immunoglobulin heavy chain (IgH) was also detected in the immunoblot.

**Fig. 3. f3:**
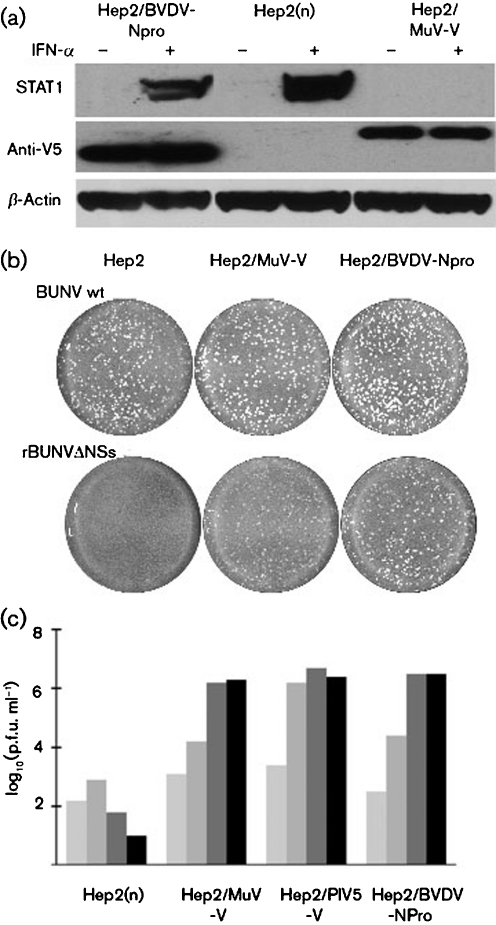
Characterization of Hep2 cells that constitutively express the V protein of MuV Enders. (a) STAT1 is degraded in cells that constitutively express MuV-V. Naïve Hep2, Hep2/BVDV-Npro and Hep2/MuV-V cells were or were not treated with IFN-*α* [Roferon A (Roche) 1000 IU ml^−1^] for 18 h and STAT1 was detected by immunoblot analysis. BVDV-Npro and MuV-V had N-terminal or C-terminal V5 tags, respectively, and their presence was detected by an anti-V5 tag antibody; *β*-actin acted as a loading control. (b) Unlike wild-type Bunyamwera virus (BUNV wt), which forms plaques in naïve Hep2, Hep2/MuV-V and Hep2/BVDV cells, a recombinant Bunyamwera virus that does not encode the NSs protein, termed rBUNVΔNSs, does not form plaques in naïve Hep2 cells but does plaque Hep2/MuV-V and Hep2/BVDV cells. Plaques shown are 4 days p.i. (c) The ability of naïve Hep2, Hep2/MuV-V, Hep2/PIV5-V and Hep2/BVDV-Npro cells to support the replication of MuV was compared. Cells were infected at an m.o.i. of 0.01 p.f.u. per cell and the amount of infectious virus in the culture medium was titrated at 2, 4, 6 and 8 days p.i. (light, medium and dark grey, and black, respectively).

One obvious explanation as to why MuV Enders replicated efficiently in ‘IFN-compromised’ Hep2 cells but poorly in naïve ‘IFN-competent’ Hep2 cells could have been that it did not encode a functional V protein. We therefore examined whether MuV Enders blocked IFN-signalling and whether the V protein interacted with mda-5. Hep2 cells were infected with MuV Enders prior to treatment with IFN, and the levels of STAT1 (which is inducible by IFN) and STAT3 were estimated by immunoblot analysis. Uninfected cells treated with IFN were used as a control. STAT1 was clearly degraded by MuV Enders in untreated cells by 8 h p.i. and the high levels of STAT1 in IFN-pretreated cells were significantly reduced by 24 h p.i. (Fig. 2a[Fig f2]), consistent with previously published work that has shown that the V protein of MuV targets STAT1 for proteasome-mediated degradation (Ulane *et al.*, 2003). Interestingly, whilst STAT3 was also degraded in untreated cells (in agreement with data from Ulane *et al.*, 2003) and although its expression is not induced by IFN, there was no evidence of STAT3 degradation at 8 h p.i. in IFN-pretreated cells, although some degradation of STAT3 was observed at 24 h p.i. Whilst the reasons for this remain unclear, it suggests that IFN-pretreatment is somehow affecting the efficiency of degradation of STAT3. Next, the MuV Enders V gene was cloned into a derivative of the plasmid pHR-SIN-CSGW that can be used either in transient transfections for expression of the gene of interest or to generate appropriate lentivirus vectors (Demaison *et al.*, 2002). 293 cells were co-transfected with plasmids expressing the FLAG-tagged helicase domain of mda-5 together with a plasmid expressing V5-tagged V protein from MuV Enders, or with a control plasmid. The V protein was immunoprecipitated with anti-V5 antibody and the immunoprecipitates were blotted for FLAG-tagged mda-5. The mda-5 helicase domain co-precipitated with the V protein of MuV Enders (Fig. 2b[Fig f2]). We next engineered a Hep2 cell line to constitutively express the MuV Enders V protein. As expected, STAT1 could not be detected in these cells even following treatment with IFN for 24 h (Fig. 3a[Fig f3]). Furthermore, they supported the replication of a number of IFN-sensitive viruses, including a recombinant Bunyamwera virus with a deletion in its NSs gene (Fig. 3b[Fig f3]), as well as MuV Enders (Fig. 3c[Fig f3]).

### MuV Enders variants that grow to high titres in naïve Hep2 cells do not have alterations in their V gene

**Fig. 4. f4:**
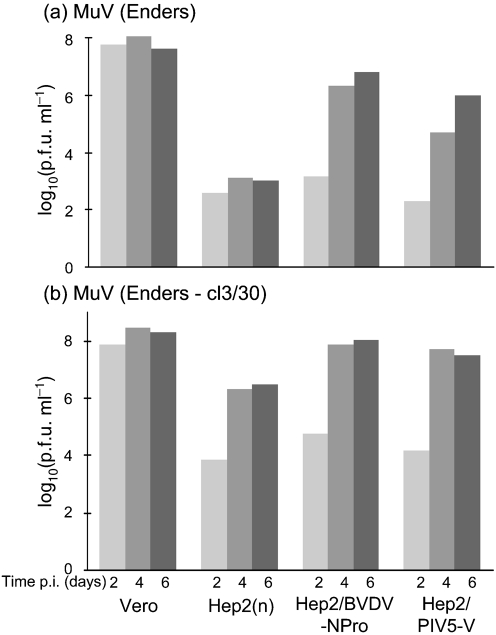
Variants of MuV Enders selected for growth on naïve Hep2 cells replicate faster both in naïve Hep2 cells and in Hep2 cells expressing PIV5-V or BVDV-Npro. The listed cells were infected at an m.o.i. of 0.01 p.f.u. per cell with either MuV Enders (a) or a subclone (cl3/30) thereof that was derived by continuous passage of MuV Enders through naïve Hep2 cells (see Methods) (b). The amount of infectious virus present in the culture supernatant was titrated at 2, 4 and 6 days p.i.

Although the MuV V protein inhibited IFN signalling and interacted with mda-5, it remained possible that to adapt to grow in naïve Hep2 cells, MuV Enders might need to improve/alter in some manner the efficacy by which its V protein acts as an IFN antagonist. To test this, variants of MuV Enders that replicated to high titres in ‘IFN-competent’ Hep2 cells were isolated and the V genes of these variants were sequenced. These variants were isolated by infecting naïve Hep2 cells with MuV Enders at an m.o.i. of ∼1 p.f.u. per cell and every 2–3 days the infected cells were passaged together with a 10-fold (passages 1–3) or 100-fold (passages 4 and 5) excess of uninfected naïve Hep2 cells. Four days after the fifth passage, virus released into the medium was subcloned on Vero cells and 30 subclones were isolated. Low m.o.i. growth curves of a number of these clones were performed on naïve Hep2, Hep2/BVDV-Npro and Hep2/PIV5-V cells. Unlike the parental MuV, these variants replicated to high titres in naïve Hep2 cells, reaching a final titre of ∼10^6^ p.f.u. ml^−1^. All subclones had similar growth curves, although only those of cl3/30 are shown as an example (Fig. 4).

To determine whether the ability of these selected subclones to grow in ‘IFN-competent’ Hep2 cells was due to mutations in their V proteins, the V genes of five subclones (including cl3/30) were cloned and sequenced. No changes resulting in amino acid substitutions were observed in the V gene sequences.

### Whole genome sequencing of MuV Enders cl3/30 reveals it has a single mutation in its HN gene

**Fig. 5. f5:**
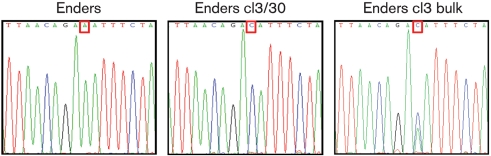
Electrochromatograms showing position 8105 in the Enders strain, the passaged cl3/30 virus and the Enders cl3 bulk. Cells were infected with the viruses, total RNA was prepared and RT-PCR was used to produce amplicons that represented the complete genome. A single coding A→C mutation was identified in the ORF of the Enders cl3/30 HN protein (HN N498H). Consensus sequencing of the bulk from which the clones were isolated indicated that this contained a mixed population. Red, T; green, A; blue, C; black, G.

To determine the genetic basis of the adaptation to growth in Hep2 cells, the complete genomes of MuV Enders and the variant cl3/30 were sequenced. The sequence of cl3/30 differed from MuV Enders at a single position (Fig. 5). Adenine at nt 8105 was mutated to cytosine, resulting in a change from asparagine to histidine at amino acid 498 of the HN protein. To determine if this was a dominant mutation in the population of viruses (bulk stock) that had been initially selected by passaging through Hep2 cells prior to subcloning on Vero cells (see above), RT-PCR and consensus sequencing of this region were performed on the bulk stock. Interestingly a double peak composed of A and C was observed at nt 8105, indicating a mixed population of both mutated and wild-type genomes. The two peaks were of equal height on the electrochromatogram, indicating that a significant proportion of viruses within the initial selected population of viruses contained the HN N498H mutation. However, it was also clear that not all viruses within the initial selected bulk population contained the asparagine to histidine substitution at amino acid 498 of the HN protein, raising the possibility that other unidentified mutations also enhance MuV Enders replication in naïve Hep2 cells.

### Variants of MuV Enders that replicate to high titres in naïve Hep2 cells can be isolated by selecting for rapidly growing viruses in IFN-compromised Hep2 cells

**Fig. 6. f6:**
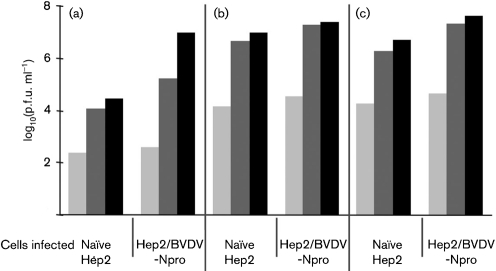
Variants of MuV Enders that replicate in naïve Hep2 cells can be isolated by selecting for rapidly replicating viruses in Hep2 cells expressing BVDV-Npro. Naïve Hep2 or Hep2/BVDV-Npro cells were infected with MuV Enders (a) or with virus that had been selected on naïve Hep2 cells (b) or on Hep2/BVDV-Npro cells (see Methods) (c) at an m.o.i. of 0.01 p.f.u. per cell. The amount of infectious virus present in the culture supernatant was titrated at 1, 2 and 4 days p.i. (light grey, dark grey and black bars, respectively).

Further comparison of the growth curves of cl3/30 revealed that it (and the other subclones tested) grew more rapidly in both Hep2/BVDV-Npro and Hep2/PIV5-V cells than the parental virus (Fig. 4). Thus, whilst in Hep2/BVDV-Npro cells, cl3/30 had reached a titre of ∼10^5^ p.f.u. ml^−1^ by 2 days p.i. and 10^8^ p.f.u. ml^−1^ by 4 days p.i., the parental virus had only reached titres of 10^3^ and 10^6^ p.f.u. ml^−1^ by 2 and 4 days p.i., respectively. To investigate whether the growth kinetics of MuV Enders influence its sensitivity to the IFN response further, we tested whether variants of MuV Enders that had been selected because they grew rapidly in Hep2/BVDV-Npro cells (i.e. without the selective pressure of IFN) would also be able to propagate in naïve Hep2 cells. A similar selection procedure (as previously described for the selection of cl3/30) was undertaken in both naïve Hep2 and Hep2/BVDV-Npro cells since during this procedure rapidly replicating viruses would have a significant advantage and would therefore be selected. As predicted, selected viruses grew faster in Hep2/BVDV-Npro cells than the parental MuV Enders (Fig. 6). Thus, whilst the titre of the parental virus on Hep2/BVDV-Npro cells was only 4×10^2^ p.f.u. ml^−1^ at 1 day p.i., the titre of the variants (selected on Hep2/BVDV-Npro cells) was 5×10^4^ p.f.u. ml^−1^, and at 2 days p.i. the titres were 2×10^5^ p.f.u. ml^−1^ and 4×10^7^ p.f.u. ml^−1^, respectively. Furthermore, these rapidly growing variants also replicated to high titres in naïve Hep2 cells (Fig. 6). RT-PCR and consensus sequencing of the HN gene, covering amino acids 436–583, was undertaken on the rapidly growing viruses selected on Hep2/BVDV-Npro cells to determine whether the asparagine to histidine substitution at amino acid 498 of the HN protein had occurred. However, this mutation was not identified, confirming that other, as-yet unidentified, mutations can also enhance virus replication sufficiently to allow MuV Enders to replicate in naïve Hep2 cells.

## DISCUSSION

We have shown that although MuV Enders encodes a functional V protein that targets STAT1 for degradation and interacts with mda-5, MuV Enders does not grow well in naïve Hep2 cells but will replicate to high titres (10^6^–10^7^ p.f.u. ml^−1^) in ‘IFN-compromised’ Hep2 cells. However, even in ‘IFN-compromised’ Hep2 cells, the propagation of MuV Enders is significantly slower than in Vero cells or when compared with the replication rate of PIV5 in ‘IFN-compromised’ Hep2 cells. This proves that although the host cell restriction that slows the growth rate of MuV Enders in Hep2 cells is independent of the IFN response, a consequence of that slower growth rate was that it enabled the IFN response of Hep2 cells to inhibit the growth of MuV Enders in multi-cycle growth assays. This conclusion is supported by the fact that variants of MuV Enders selected by passage through naïve Hep2 cells replicated more rapidly than MuV Enders in ‘IFN-compromised’ Hep2 cells. To test directly whether viral growth kinetics could influence the sensitivity of MuV to the IFN response, we isolated variants of MuV Enders that could grow rapidly in ‘IFN-compromised’ Hep2 cells and then examined the replication of these viruses in naïve Hep2 cells. As predicted, these variant viruses with faster growth rates now replicated to high titres in naïve Hep2 cells, even though they had been selected in the absence of any selective pressure exerted by the IFN response, highlighting the usefulness of such a forward genetics approach. Importantly, this also supports the idea that there is a race between the ability of cells to detect and respond to virus infections and the ability of a virus to block the IFN response (Randall & Goodbourn, 2008).

Work on other viruses also suggests that factors other than the property of the viral IFN antagonists can influence whether viruses can replicate in ‘IFN-competent’ cells (Young *et al.*, 2003). Thus, it is clearly important for viruses to control their production of PAMPs during their replication cycle. For example, it has been reported that although the C protein of measles virus (MeV) has no intrinsic activity that blocks the IFN induction pathway, it acts as a regulator of viral RNA synthesis, thereby acting indirectly to suppress IFN induction, and hence influences the sensitivity of MeV to the IFN system (Nakatsu *et al.*, 2008). Similarly, work on PIV5 has also suggested that in order to limit IFN production, viruses must control the balance of their transcription and replication in order to limit the levels or types of RNA that induce the production of IFN (Dillon & Parks, 2007; Timani *et al.*, 2008). Results presented here on whole-genome sequencing of one variant (cl3/30) of MuV Enders that could replicate in naïve Hep2 cells revealed that it differed from the parental strain at a single strictly conserved position in the HN gene in a highly conserved region of the protein. As this was the only change in the entire genome, it is reasonable to assume that this mutation is responsible for the more rapid growth rate of cl3/30, and thus suggests that the entry and egress of viruses may also (indirectly) influence their sensitivity to the IFN response. However, it should also be noted that sequence analysis of the bulk population of viruses selected on naïve Hep2 cells prior to subcloning revealed that not all the viruses had the asparagine to histidine substitution at amino acid 498 of the HN protein. Furthermore, this mutation was not identified in rapidly growing viruses selected on Hep2/BVDV-Npro cells, confirming that other, as yet unidentified, mutations can also enhance the speed of replication of MuV Enders sufficiently for it to be able to replicate in naïve Hep2 cells. Quasi-species compositions of viral populations that are not tractable by consensus sequencing of RT-PCR products have been identified as virulence determinants in foot and mouth disease virus (Sanz-Ramos *et al.*, 2008), further complicating interpretations of the meaning of single nucleotide changes in RNA viruses. Hence, we are currently developing reverse genetics for MuV Enders in order to address fundamental questions as to the underlying molecular reasons why MuV Enders grows poorly in Hep2 cells. In general, it will be of interest to ascertain whether mutations that slow the growth cycle of viruses at any point from virus entry to exit can sensitize them to the IFN response. If this is the case, it cannot be concluded that viruses (wild-type or mutant) that cannot grow in ‘IFN-competent’ cells or animals but can replicate in ‘IFN-compromised’ derivatives must have a defect in their IFN antagonists. In this regard, it is of note that insertion of the ORF of enhanced green fluorescent protein into the major component of the viral RNA-dependent RNA polymerase of measles virus, rinderpest virus and canine distemper virus reduces the replication rate of these viruses to such a degree that they are unable to overcome the host's antiviral defences mechanisms and are thus attenuated *in vivo* (Brown *et al.*, 2005; Plumet *et al.*, 2005; Silin *et al.*, 2007). Furthermore, it has been reported that for influenza A viruses to be highly pathogenic in Mx1-positive strains of mice, as well as encoding a functional IFN antagonist, they also need to have a very rapid replication rate in order to out-compete the antiviral response of the host (Grimm *et al.*, 2007). An understanding of the factors that influence the growth rates of viruses, and the effects these factors have on the sensitivity of viruses to the IFN system, will be important in order to appreciate virus tropism and pathogenicity fully.
